# Production of Plant-Based Seafood: Scallop Analogs Formed by Enzymatic Gelation of Pea Protein-Pectin Mixtures

**DOI:** 10.3390/foods11060851

**Published:** 2022-03-17

**Authors:** Zhiyun Zhang, Kanon Kobata, Hung Pham, Dorian Kos, Yunbing Tan, Jiakai Lu, David Julian McClements

**Affiliations:** 1 Department of Food Science, University of Massachusetts, Amherst, MA 01003, USA; zhiyunzhang@foodsci.umass.edu (Z.Z.); kkobata@umass.edu (K.K.); hvpham@umass.edu (H.P.); dkos@umass.edu (D.K.); ytan@umass.edu (Y.T.); jiakailu@umass.edu (J.L.); 2 240 Chenoweth Laboratory, 102 Holdsworth Way, Amherst, MA 01003, USA

**Keywords:** plant-based foods, pea protein, pectin, thermodynamic incompatibility, transglutaminase

## Abstract

This study investigated the possibility of using a phase separation, mixing, and enzymatic gelation approach to construct seafood analogs from plant protein-polysaccharide mixtures with properties mimicking real seafood. Heat-denatured pea protein (10%, *w*/*w*) and pectin (0–1%, *w*/*w*) were mixed to produce phase separated biopolymer blends. These blends were then subjected to mild shearing (350 rpm) to obtain fiber-like structures, which were then placed in molds and set by gelling the pea proteins using transglutaminase (2%, *w*/*w*). The appearance, texture, and cooking properties of the resulting scallop analogs were characterized and compared to those of real scallop. The presence of the pectin promoted the formation of a honeycomb structure in the scallop analogs, and microscopic orientation of the proteins was observed in the plane parallel to the applied shear flow. Lower pectin concentrations (0.5%, *w*/*w*) led to stronger gels with better water holding capacity than higher ones (1.0%, *w*/*w*). The appearance and texture of the plant-based scallop analogs were like those of real scallop after grilling, indicating the potential of using this soft matter physics approach to create plant-based seafood analogs. One of the main advantages of this method is that it does not require any expensive dedicated equipment, such as an extruder or shear cell technology, which may increase its commercial viability.

## 1. Introduction

Consumers are increasing the number of plant-based foods in their diet due to environmental, health, and animal welfare concerns, including meat, seafood, egg, and dairy alternatives [1,2,3]. In this study, we focused on the development of a model plant-based seafood, as there is currently a lack of high-quality products in this area [4]. Seafood is an important source of protein in the human diet, as well as a good source of other health-promoting nutrients, such as omega-3 fatty acids, vitamins, and minerals. However, over-exploitation of wild seafood populations is depleting the oceans of these valuable resources [5,6]. Moreover, climate change is altering fish migration patterns, with profound effects on the fishing industry and coastal communities [7,8]. Wild seafood may also contain appreciable levels of toxins, especially mercury, persistent organic pollutants, and microplastics, which adversely affect human health [9,10,11]. Seafood extraction and processing have also been reported to be a significant contributor to greenhouse gas (GHG) emissions [12]. Finally, seafood, such as fish and shellfish, are a major source of allergens to a significant fraction of the population. The rapidly growing aquaculture industry alleviates some of these issues, but has its own challenges, including the need for protein-rich resources to feed the fish, as well as its propensity to cause pollution, such as eutrophication [13,14]. There are also substantial losses in aquaculture due to diseases, such as sea lice in farmed salmon, which contribute to food waste and economic losses estimated to be around $6 billion per year [13]. Moreover, there are concerns that the antibiotics and pesticides used to tackle these diseases may contaminate fish and the environment. The availability of plant-based seafood analogs would help to reduce many these of these problems by creating an alternative to real seafood, thereby allowing existing seafood stocks to be managed more sustainably [15].

Plant-based foods have been fabricated using several processing technologies, including extrusion, shear cell, spinning, and 3D printing methods [16,17,18,19,20,21,22,23]. At present, extrusion is the most commonly used technology for the industrial production of plant-based foods because of its simplicity, versatility, and scalability [2,24]. In this approach, plant-based materials (usually proteins and polysaccharides) are heated and sheared under high pressure in a device that contains a barrel with a series of screws to mix and transport the materials. These processes change the solubility, conformation, and interactions of the proteins, which promotes the formation of protein aggregates. These aggregates are aligned in the direction of flow when the material passes through a long cooling die attached to the end of the extruder, leading to the creation of an anisotropic food matrix with meat-like structures and textures [2,25]. The shear cell technology also has potential to produce plant-based foods on a commercial scale [26,27]. Indeed, it has recently been adopted by a start-up company (Rival Foods) to produce plant-based meat analogs. This device has a cylinder-in-cylinder design, which consists of a heated stationary outer cylinder with a lid and a heated inner cylinder that is rotated via a drive shaft. Raw samples are pre-mixed and placed in the gap between the two cylinders. Unlike extrusion, the material deformation inside the shear device is well controlled and constant during the manufacturing process [26,27]. However, both extrusion and shear cell technologies require specialized equipment and high energy inputs, which limits their suitability for smaller companies and leads to some environmental concerns. A simpler, cheaper, and more energy-efficient means of creating plant-based meat and seafood products would therefore be advantageous.

Phase separation of protein-polysaccharide mixtures due to thermodynamic incompatibility can be used to create novel microstructures and textures in foods [28,29]. This approach is based on the fact that the free energy of a phase separated mixture of two types of biopolymers that repel each is lower than that of an intimate mixture [2]. The tendency for phase separation to occur is influenced by several factors, including the type and concentration of the biopolymers, as well as the pH and ionic strength of the surrounding solution [30]. After phase separation, the mixed biopolymer system can be stirred to form a “water-in-water” (*w*/*w*) emulsion, which consists of a dispersed phase rich in one kind of biopolymer and a continuous phase rich in the other kind of biopolymer. The droplets in *w*/*w* emulsions are characterized by a very low interfacial tension, which means they can be easily deformed and elongated into fiber-like structures by applying low shear stresses [18]. These structures can then be locked into place by promoting gelation of the dispersed and/or continuous biopolymer phase. This soft matter physics approach can therefore be used to create foods with meat-like structures and textures from plant proteins and polysaccharides.

In this study, the thermodynamic incompatibility approach was used to create seafood (scallop) analogs from plant proteins and polysaccharides. Commercial sea scallop analogs are already available, but most of them use fish or whey proteins as structuring agents. Those plant-based scallops that are on the market have protein concentrations (<2.5%) considerably below those of real scallops (10% to 12%), e.g., those sold by the Plant Based Seafood Company. These products tend to use starches and gums as structuring agents rather than proteins. In our study, we used pea protein and high methoxy citrus pectin as the protein and polysaccharide to formulate the scallop analog. These biopolymers were chosen because pea protein is not a major allergen and citrus pectin is a dietary fiber. Moreover, pea protein is a highly functional and affordable protein that can be obtained in sufficiently large quantities for commercial production. Similarly, citrus pectin is already widely used as a functional ingredient in the food industry and is also available at sufficiently large quantities for commercial applications. The pea protein concentration was chosen to be close to that of a real scallop to match its nutritional content. The two biopolymers were mixed and blended to promote phase separation and fiber formation, placed in a mold, and then the pea proteins were crosslinked using a food-grade enzyme (transglutaminase) to lock the fiber structures in place and increase the gel strength. The structural and physicochemical properties of the plant-based scallops produced by this method were then compared to those of real sea scallops, including their microstructure, color, texture, water holding capacity, and cookability. To the best of our knowledge, this is the first study that has used a soft-matter physics approach to construct plant-based scallop analogs. One of the major potential advantages of this approach over existing methods of creating plant-based foods is that no expensive and energy-intensive structuring equipment is required, such as an extruder or a shear cell. Consequently, it may have considerable commercial potential for the production of these kinds of products. Moreover, it should be possible to use other combinations of proteins and polysaccharides to create these kinds of plant-based foods, which would lead to considerable flexibility in sourcing ingredients.

The knowledge gained from this study could be used by the food industry to create plant-based seafood analogs with improved quality, nutritional profile, and cooking properties. The availability of these products could facilitate the transition to a more sustainable and environmentally friendly food supply.

## 2. Materials and Methods

### 2.1. Materials

Native yellow pea flour was provided by Prof. Jiajia Rao, from North Dakota State University. Pectin from citrus peel (galacturonic acid ≥ 74.0% dried basis) was purchased from Sigma-Aldrich Co., Ltd. (St. Louis, MO, USA). ACTIVA RM transglutaminase (T-gase) preparation was purchased from Ajinomoto North America., Inc. (Chicago, IL, USA). Raw sea scallops were bought from a local grocery store (Stop & Shop, Amherst, MA, USA) and stored in a freezer (−20 °C) until used. Sodium hydroxide (NaOH) and hydrochloric acid (HCl) were purchased from Fisher Scientific (Waltham, MA, USA). The Bradford reagent used for the protein determination was obtained from the Bio-Rad company (Hercules, CA, USA).

### 2.2. Protein Extraction

Pea protein isolate (PPI) was extracted from yellow pea flour according to a method described previously, with some modifications [31]. Briefly, yellow pea flour (100 g) was dissolved in 1500 g of deionized water, and the solution was then adjusted to pH 9.0 using 6 N of NaOH. The alkaline protein solution was then continuously stirred using a magnetic stirrer at 500 rpm for 1 h at room temperature. The pH was checked every 15 min and adjusted back to 9.0 if necessary. Then, the solution was centrifuged at 5524× *g* for 20 min at 4 °C. The supernatant was filtered through a Whatman grade 1 (Whatman Grade 42, ashless, 90 mm diameter) using a bench-top vacuum and collected in a flask that was cooled down in an ice bath. The supernatant was then adjusted to pH 4.5 using 6 N of HCl followed by centrifugation at 5524× *g* for 20 min at 4 °C. The pellet from centrifugation was collected and re-suspended in water, and the solution was adjusted back to pH 7.0 using 1 N of NaOH. Powdered PPI was obtained by freeze-drying the pellet solution for 48 h.

### 2.3. Extracted Protein Concentration

The concentration of extracted pea protein was determined by the Bradford protein assay [32]. In brief, a standard curve was prepared using a series of bovine serum albumin (BSA) solutions of different protein concentrations (0 to 1000 µg/mL). For the test samples, 20 *w*/*w*% of pea protein stock solution was diluted 1000 times with deionized water. Then, 20 µL of diluted pea protein solution was vortex-mixed with 1 mL of Bradford reagent, incubated for 10 min, and the absorbance was measured at 595 nm using UV-visible spectrometer. The protein concentration was then estimated from the standard curve. The test samples were prepared in duplicates and the blank consisted of deionized water. The protein concentration of the stock solution was assessed every time after overnight rehydration.

### 2.4. Differential Scanning Calorimetry Analysis

The thermal transitions of pea proteins dissolved in aqueous solutions were assessed by measuring changes in the heat flow with temperature using a differential scanning calorimeter (DSC 250, TA Instruments, New Castle, DE, USA). Pea protein solutions (20 *w*/*w*%) were placed in a high-volume aluminum pan that was then tightly sealed. Another empty high-volume aluminum pan was used as a reference. The weight of each test sample used in the DSC analysis was recorded. DSC measurements were performed by heating the samples from 10 to 130 °C at 3 °C/min under an inert atmosphere (400 mL/min of N2). The onset temperature (To), peak temperature (Tp), and enthalpy (∆H) of the transitions were computed from the thermal curves using the instrument software (TRIOS 5.2). The same samples were then heated again under the same conditions to establish whether the thermal transitions were reversible.

### 2.5. Pea Protein-Pectin Gel (Scallop Analog)

Extracted pea proteins were rehydrated overnight to prepare 20 *w*/*w*% pea protein stock solutions. These stock solutions were then diluted to 10 *w*/*w*%, and the pH was adjusted back to 7.0. Ten grams of pea protein solution were dispensed into a 15 mL beaker (used as a scallop-shaped mold) and then heat-denatured and aggregated by holding at 95 °C for 30 min. This procedure was carried out to increase the effective molecular weight of the proteins, thereby reducing the entropy of mixing effects in the subsequent biopolymer mixtures. After cooling the heat-denatured pea proteins in an ice bath for another 30 min, different concentrations of pectin (0, 0.5, or 1.0 w_pectin_/w_total_%) were added and the mixtures were stirred at 500 rpm at room temperature for 60 min to ensure dissolution. Then, 2.0 w_T-gase_/w_total_% of transglutaminase (T-gase) was added to the biopolymer mixtures and the system was stirred for 30 min at 500 rpm at room temperature to promote enzyme dissolution. The stir bar was then removed, and the samples were incubated at 50 °C for 30 min to promote protein crosslinking, followed by 30 min of cooling in an ice bath. The gels formed were then gently removed from the beakers and placed onto petri dishes.

### 2.6. Fourier-Transform Infrared Spectroscopy (FTIR) Analysis

FTIR spectra were acquired using a Fourier Transform Infrared spectrophotometer (Shimadzu, Kyoto, Japan) equipped with an attenuated total reflectance (ATR) accessory under ambient conditions. The samples analyzed by the ATR-FTIR instrument were prepared according to a method described previously [33]. Briefly, freeze-dried powdered pea protein, pectin, or pea protein-pectin (uncooked scallop analog) were placed between two pieces of aluminum foil and then pressed into a small pellet. This pellet was then further pressed onto the germanium crystal surface using an ATR accessory to ensure good contact with the ATR crystal. The background signal was collected before each measurement. Each spectrum was the average of 32 scans in the wavenumber range from 4000 to 400 cm^−1^ at a 4 cm^−1^ resolution.

### 2.7. Texture Profile Analysis

A texture analyzer (TA.XT2, Stable Micro System, Surrey, UK) with a flat-ended cylinder probe (25 mm diameter) was used to characterize the mechanical properties of the scallop and scallop analog. Double compression was applied to all the samples and the texture profile analysis (TPA) parameters were calculated from the resulting stress-strain curves based on the methods described in a previous study [34]. In brief, a cylindrical test sample of fixed dimensions (4 cm diameter × 0.8 cm height) was placed on the instrument lower plate and the measurement probe was moved downward at a pre-speed of 2 mm/s. When the probe first touched the surface of the test samples, their thickness was automatically recorded. The probe continued to press the samples to a final strain of 50% at a test speed of 2 mm/s. Then, the samples were allowed to recover for 15 s by removing the force of the probe that was applied on their surfaces. After that, the probe was then pressed onto the samples again, which resulted in a double compression, and then returned to its original position at a post-test speed of 2 mm/s. The trigger force was set to 0.049 N (5 g). The following parameters were then calculated from the texture analysis (TPA) profiles of each sample [34]:

Hardness: The hardness is a measure of the resistance of the sample to compression, which was taken to be the maximum force reached during the first compression of the sample (Fmax1).

Cohesion: The cohesion is a measure of how well the sample maintains its textural attributes after the first deformation, which was calculated as the ratio of areas under the curves for the second and first peaks in the TPA profile (A2/A1).

Springiness: The springiness is a measure of how well the sample springs back to its original dimensions after it has been deformed using a first compression, allowed to sit for 15 s, and then deformed again using a second compression. It is calculated as the ratio of the distances from the start of compression until the maximum is reached for peak 2 and peak 1 (D2/D1).

Chewiness: The chewiness is a measure of the energy required to chew solid foods, which is calculated as, Chewiness = Hardness × Cohesion × Springiness.

### 2.8. Scanning Electron Microscope Analysis

Both scallop and scallop analog were freeze-dried (Genesis Pilot Lyophilizer, SP Scientific, Stone Ridge, NY, USA) and then sputter-coated with gold [35]. All samples were examined by scanning electron microscopy (SEM) using a FEI Magellan 400 (FEI, Hillsboro, OR, USA) with an accelerating voltage of 5 kV under low vacuum conditions.

### 2.9. Other Physical and Functional Parameters

#### 2.9.1. Water Holding Capacity

The water holding capacity of both scallops and scallop analogs were analyzed using a centrifugal method. A fixed amount (0.50 g) of each initial test sample was placed into a centrifuge tube and then centrifuged at 10,000 rpm at room temperature for 15 min. Any water released from the test samples was carefully removed using a pipette and their final weight was measured. The water holding capacity was calculated as follows:

WHC (%) = (Initial Weight [g])/(Final Weight [g]) × 100(1)

#### 2.9.2. Colorimetric Analysis

The tristimulus color coordinates (L*, a*, b*) of the real scallop and scallop analog were measured using a colorimeter (ColorFlez EZ, HunterLab, Reston, VA, USA). The L* value describes lightness, the a* value describes redness/greenness, and the b* value describes blueness/yellowness (Commission Internationale de l’Eclairage, Vienna, Austria).

#### 2.9.3. Cookability

The impact of pan frying on the structural and physicochemical properties of the real scallop and scallop analog was also tested. The samples were placed in a non-stick frying pan and heated on each side for 3 min, leading to a total cooking time of 6 min. The internal temperature was monitored with a 0.1 mm diameter copper-constantan thermocouple (Type-T). After cooking, the microstructure, texture and color of the scallop and scallop analog were measured.

### 2.10. Statistical Analysis

Triplicate analyses were performed for all measurements. Statistical analysis was conducted using Microsoft Excel 2019 software to determine *p* values using a student’s *t* test. Significant differences (*p* < 0.05) between different group means were determined with the Tukey-Kramer HSD test.

## 3. Results and Discussion

### 3.1. Characterization of Extracted Pea Proteins

**Figure 1:**
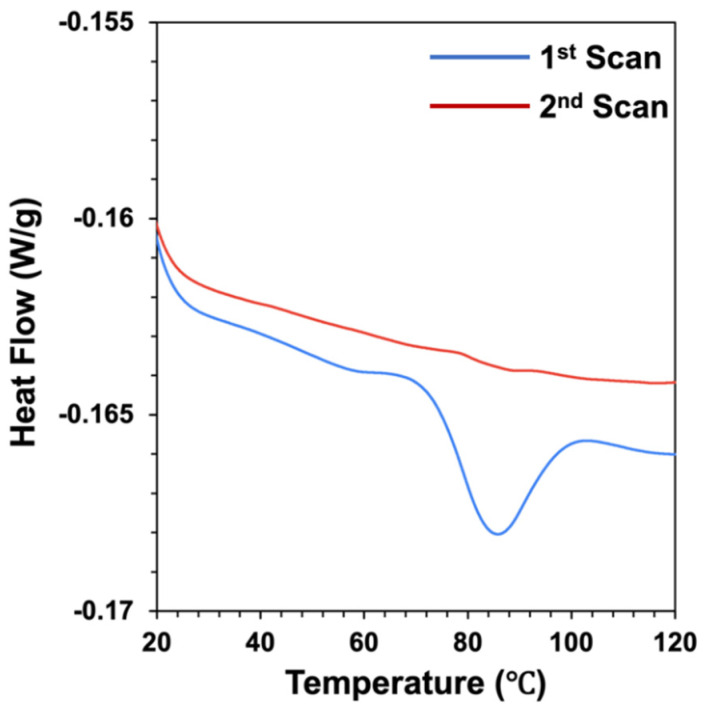
Heat flow versus temperature profiles of pea protein (20 wt%) when heated twice from 10 to 120 °C at 3 °C min^−1^. The endothermic peak observed during the first scan suggests the proteins were originally in the native state, whereas the lack of peaks in the second scan suggests that they were irreversibly denatured by heating.

The molecular state of the extracted pea proteins (native or denatured) was determined using differential scanning calorimetry. The heat flow versus temperature profiles of pea protein solutions (20% *w*/*w*%) were measured when they were heated from 10 to 120 °C at a heating rate of 3 °C/min (Figure 1). The same sample was then cooled and heated again under the same conditions to establish whether any observed thermal transitions were reversible or irreversible. During the first scan, a major peak was observed at a temperature (Tpeak) around 85 °C, which was associated with an endothermic enthalpy change (H) of around 1.02 J/g. This endothermic peak was attributed to the thermal denaturation of the globulin fraction of the pea protein. Similar thermal denaturation temperatures have been reported for globulin pea proteins in other studies, e.g., 88 °C [36] and 86 °C [37]. Some researchers have reported two endothermic peaks for pea protein isolates during heating: one corresponding to the denaturation of the non-globulin fraction (around 67 °C) and another corresponding to the denaturation of the globulin fraction (around 85 °C) [38]. During the second scan of the pea protein solution, we found that the peak associated with the thermal denaturation of the proteins was greatly diminished (Figure 1), which suggested that most of the protein molecules had been irreversibly denatured during the first scan.

### 3.2. Preparation of Scallop Analogs

**Figure 2:**
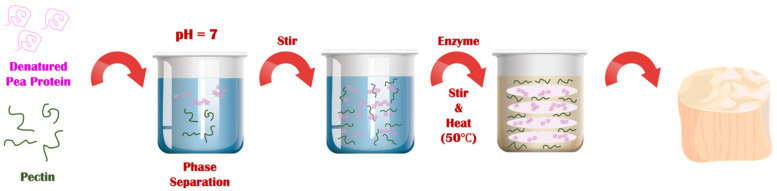
Scallop analogs can be formed through controlled phase separation, shearing, and gelling of mixtures of heat-denatured pea proteins and pectin.

The series of steps used to prepare the plant-based scallops is shown schematically in Figure 2. Each major step in the process is described here with a discussion of the underlying physicochemical principles:

First, a solution of native pea proteins was heated (95 °C, 30 min) above its thermal denaturation temperature to promote the unfolding and aggregation of the protein molecules. This step is required to increase the effective molecular weight of the proteins, thereby reducing the entropy of mixing effect that opposes phase separation. This step must be carried out under appropriate protein concentration, pH, and ionic strength conditions to ensure that the protein aggregates formed have appropriate dimensions. We found that heating a 10% (*w*/*w*) protein solution at neutral pH in the absence of salt was sufficient to achieve this goal. After formation, the solution of heat-denatured proteins was cooled to room temperature. The resulting solution was more viscous than the original solution but did not gel, which suggests that protein aggregates had been formed but they were not so large that they formed a network that occupied the entirety of the system.

Second, pectin was added to the heat-denatured protein solution at room temperature, and the system was mixed. In this study, we used a pea protein concentration of around 10% to mimic the protein concentration found in real scallop (10 to 12%). Several pectin concentrations (0, 0.5, and 1.0%) were used to assess the impact of polysaccharide addition on the microstructure and textural attributes of the scallop analogs. At sufficiently high biopolymer concentrations, Phase separation of mixed protein-polysaccharide systems is known to occur under similar biopolymer concentrations due to a phenomenon known as thermodynamic incompatibility [29,39,40].

Third, when a phase separated mixed biopolymer system is gently stirred, it forms a water-in-water (*w*/*w*) emulsion, in which the disperse phase droplets are enriched in one kind of biopolymer and the continuous phase is enriched in the other kind of biopolymer. Typically, the interfacial tension at the water-water interface is relatively low (~10^−7^ to 10^−5^ N/m), which means that the droplets are easily deformed and elongated by applying relatively mild shear forces [18]. This phenomenon has previously been used to create fibrous structures from soy protein/pectin mixtures by shearing them at high temperatures in a specialized shear cell device [17,18]. The biopolymer composite material formed consisted of pectin filaments embedded within a protein matrix. We therefore postulated that fiber-like structures would also be formed in the pea protein/pectin blends used in our study when the biopolymer mixture was sheared, which was supported by our microstructural analysis (see later).

Fourth, once the fiber-like structures were formed in the biopolymer mixture, they were locked into place by gelling the proteins using 2% transglutaminase. This food-grade enzyme induces protein crosslinking by catalyzing an acyl-transfer reaction between a γ-carbonyl group of a glutamine residue and an ε-amino group of a lysine residue [41,42]. It should be noted that microbial transglutaminase is widely used in the food industry as a crosslinking agent due to its relatively low cost and “Generally Recognized As Safe” labeling status [42,43].

Fifth, the scallop analogs formed by this process were removed from the glass beakers. These beakers were selected because they had similar dimensions to real scallops and could therefore be used as molds. In industry, molds with specific seafood-like shapes and sizes could be used to form other kinds of seafood.

### 3.3. Fourier-Transform Infrared Analysis

**Figure 3:**
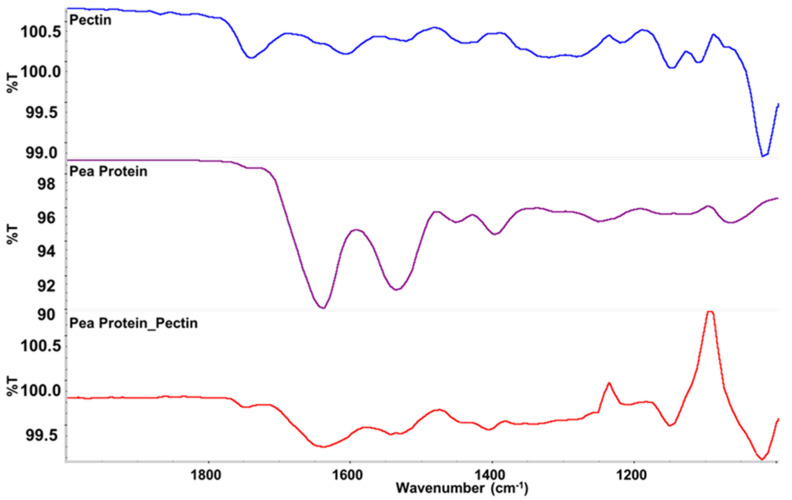
FTIR spectra of powdered pectin, pea protein, and scallop analogs. The scallop analogs consisted of 10% pea protein, 2% transglutaminase, and 0.5% pectin.

FTIR spectroscopy was used to provide information about the composition of the scallop analogs. As shown in Figure 3, bands were observed at wavenumbers of 1633, 1529, and 1389 cm^−1^, which were consistent with the C=O, N–H, and C–N stretching/bending vibrations in amide I, II and III, respectively [44]. These bands were seen in both the pure pea protein and in the scallop analogs, which confirmed that the proteins were present within the scallop analogs. The strong band observed at 1012 cm^−1^ can be assigned to intermolecular hydrogen bonding of the pectin backbone [44]. This band was seen in both the pure pectin and the scallop analogs, which confirmed that the pectin was also present within the scallop analogs. Some new peaks were observed in the spectra of the scallop, which may have been due to the presence of water or due to changes in the molecular interactions in the system when the protein and polysaccharide molecules were mixed.

### 3.4. Textural and Water Holding Properties of Scallop and Scallop Analogs

Texture profile analysis was used to provide information about the impact of product formulation on the textural attributes of the plant-based scallops, as well as to compare their textural attributes to those of real scallops.

**Figure 4:**
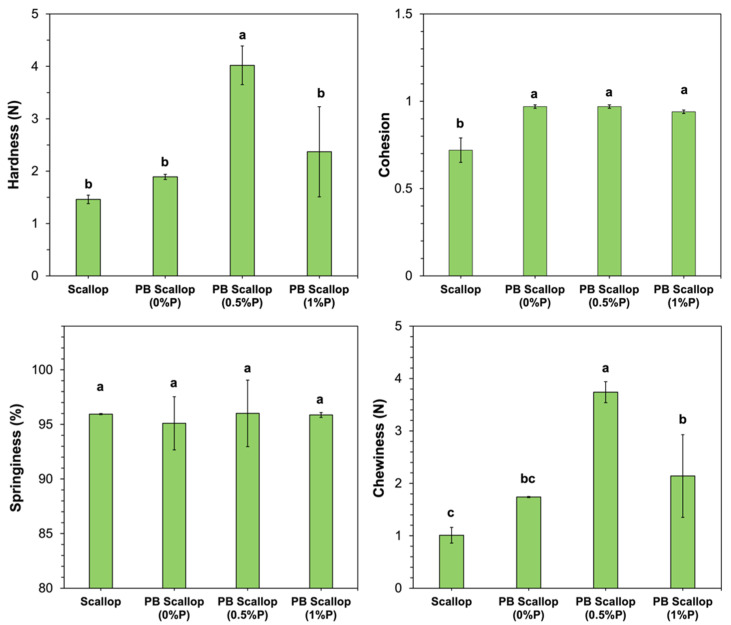
Textual profile analysis of raw scallop and uncooked scallop analogs containing different pectin concentrations. All scallop analogs contained 10% pea protein and 2% transglutaminase. Error bars represent the standard errors (*n* = 3), and similar letters mean no statistical difference between treatments (*p* ≤ 0.05).

As shown in Figure 4, the hardness, springiness, and chewiness of the scallop analog constructed from 10% pea protein, 0% pectin, and 2% transglutaminase were not statistically different from those of real scallop. However, the cohesion of these scallop analogs was significantly higher than that of the real scallops. The hardness and chewiness of the scallop analogs increased when the pectin concentration was raised from 0 to 0.5% (*w*/*w*) but then decreased when it was further raised to 1.0% (*w*/*w*). These results suggest that low concentrations of pectin strengthened the texture of the uncooked scallop analogs, while high concentrations weakened it. We postulate that low pectin concentrations may have promoted phase separation of the protein-polysaccharide mixture, which increased the protein concentration in the continuous phase, thereby strengthening the gel matrix. Conversely, high pectin concentrations may have inhibited the molecular interactions between neighboring protein molecules. Similar effects have been reported in some other studies on protein-polysaccharide mixtures. For example, a study on ginkgo seed protein-pectin composite gels found that adding relatively low pectin concentrations (<0.5% *w*/*w*) strengthened the gels but adding a higher concentration (1% *w*/*w*) weakened them [45]. The springiness of the scallops and scallop analogs was relatively high (>95%) and did not depend on the pectin concentration used (Figure 4). This latter effect suggests that the incorporation of the pectin did not affect the ability of the scallop analogs to return to almost their original dimensions after the first compression.

**Figure 5:**
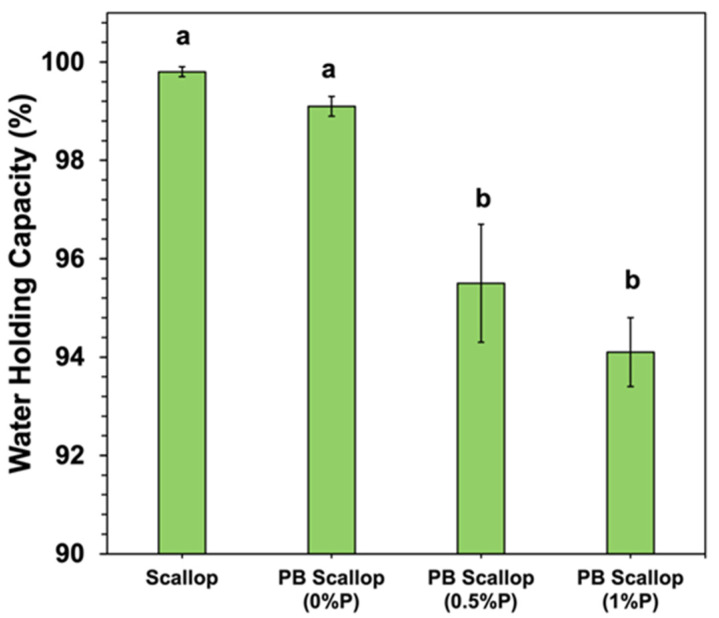
Water holding capacity of scallops and scallop analogs containing different pectin concentrations. All scallop analogs contained 10% pea protein and 2% transglutaminase. Error bars represent the standard errors (*n* = 3), and similar letters mean no statistical difference between treatments (*p* ≤ 0.05).

The water holding capacity (WHC) of the scallop and scallop analogs was also measured (Figure 5). The WHC of the scallop and scallop analog containing no pectin were quite similar, with no significant difference between them. The WHC of the scallop analogs decreased significantly with increasing pectin concentration, going from around 99.1% at 0% pectin to 94.1% at 1.0% pectin. In general, the WHC is a measure of the ability of a material to retain water when an external stress is applied, such as centrifugation [46,47]. The ability of porous food matrices to retain water can be attributed to the presence of a 3D network of entangled and crosslinked biopolymer molecules. Three main physicochemical processes typically contribute to the water holding properties of porous food matrices: (i) biopolymer-water mixing effects; (ii) ion distribution effects; and (iii) elastic deformation effects [47,48]. The biopolymer-water mixing effect depends on changes in the molecular interactions and entropy of the biopolymer and water molecules when they are combined. Consequently, it is governed by the type of molecular interactions (e.g., electrostatic, hydrogen bonding, and/or hydrophobic interactions) and contact area between the biopolymer and water molecules (which depends on the pore size of the biopolymer network). The ion distribution effect is mainly a result of concentration gradients between mineral ions inside and outside the biopolymer network, as this generates an osmotic pressure. These effects are therefore impacted by the tendency for counter-ions to accumulate around oppositely charged groups on the surfaces of biopolymer molecules in the gel network. The elastic deformation effect results from the mechanical resistance of the biopolymer network to compression when an external force (such as centrifugation) is applied: the stronger the gel network, the greater the WHC.

The observed decrease in WHC with increasing pectin concentration therefore suggests that the presence of the polysaccharide impacted one or more of these physicochemical mechanisms. The TPA measurements showed that the addition of pectin increased the hardness of the scallop analogs (Figure 4), which suggests that elastic deformation effects were not responsible for the reduction in WPC. The presence of the pectin molecules increased the pore size of the biopolymer network (see later), which would have decreased the contact area between the protein molecules and water, thereby reducing the ability of the scallop analogs to retain water. The presence of the pectin may also have altered the balance of mineral ions inside and outside the gels, which would have altered the magnitude of the osmotic stress acting on the gels, thereby altering their WHC. Nevertheless, further research is needed to identify the precise physicochemical origin of these effects.

### 3.5. Microstructure

**Figure 6:**
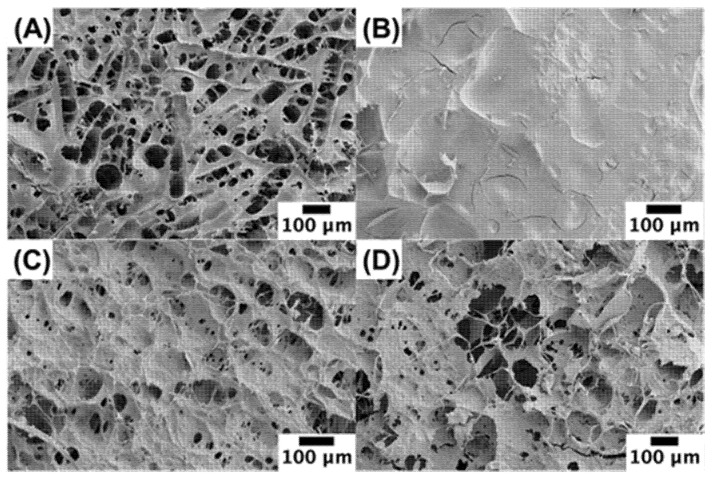
Scanning electron microscopy images of scallop (**A**) and scallop analogs containing 0% (**B**), 0.5% (**C**) and 1.0% (**D**) pectin. All scallop analogs contained 10% pea protein and 2% transglutaminase.

Scanning electron microscopy was used to provide insights into the microstructure of the scallops and scallop analogs (Figure 6A). The real scallop had a honeycomb structure, which is consistent with that reported previously for scallop adductor muscles [49]. Presumably, this structure was due to the presence of the muscle fibers in the scallop. During the dehydration process required to prepare the samples for SEM analysis, the fibers in the scallops may have separated from each other. In the absence of pectin, the scallop analogs had a much smoother microstructure than the real scallop (Figure 6B), which may have been because they only contained a network of closely packed globular pea protein molecules. As the pectin concentration was raised, the biopolymer network became more porous, and the pore size increased (Figure 6B–D). This effect may be due to the ability of the pectin molecules to promote phase separation of the pea protein-pectin mixtures, thereby leading to the formation of fiber-like structures when they were sheared during the formation of the scallop analogs. The increase in pore size with increasing pectin concentration would account for the reduction in WHC when the pectin concentration was raised (Section 3.4). Overall, these results show that the microstructure of the scallop analogs is closer to that of the real scallops when pectin is incorporated into the system.

### 3.6. Color and Textural Properties of Scallop and Scallop Analog after Grilling

In these experiments, we compared the color and textural attributes of scallop analogs to those of real scallops after grilling. Scallop analogs containing 10% pea protein, 0.5% pectin, and 2% transglutaminase were selected for these studies because they had microstructures somewhat like real scallops. Moreover, the changes in physicochemical properties caused by grilling led to final products with textural attributes more like those of real scallops.

**Figure 7:**
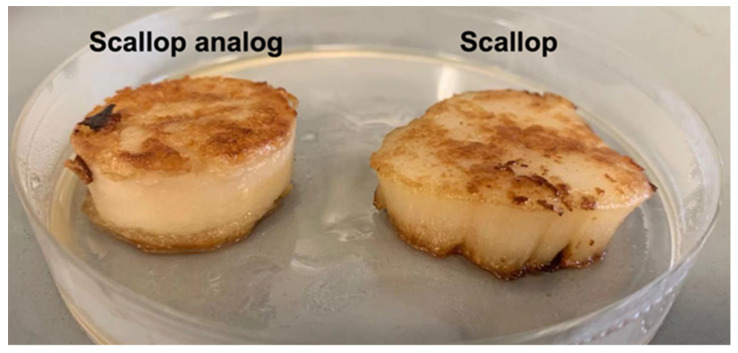
Scallop and scallop analog after grilling. The scallop analogs contained 10% pea protein, 0.5% pectin, and 2% transglutaminase.

The surfaces of the real scallop turned golden brown after grilling for 3 min on each side (Figure 7). This color change can be attributed to the Maillard reaction, which is a complex series of non-enzymatic reactions between the ϵ-amino groups of proteins and the carbonyl groups of reducing sugars. The Maillard reaction is known to occur when fish, shellfish, shrimp, and squid are thermally processed, resulting in desirable flavors and colors during cooking [50,51]. Similar to real scallops, the surfaces of the plant-based scallops also became golden brown after grilling (Figure 7), which can be attributed to a Maillard reaction between the pea protein and pectin [52].

Further information about the appearance of the grilled scallops was obtained by colorimetric analysis (Table 1). There were no significant differences between the lightness (L* value), redness (a* value), and yellowness (b* value) of the real scallops and the scallop analogs. Both types of products had intermediate lightness values (53–55), moderate redness values (24–26), and low yellowness values (0.8 to 0.9). These results suggest that the appearance of real scallops could be closely matched using the plant-based scallop analogs developed in this study.

The textural attributes of the real scallops and scallop analogs were also measured and compared after grilling. Compared to the uncooked versions, there was a large increase in the hardness and chewiness of both types of scallops after grilling. For instance, the hardness increased from 1.46 to 19.6 N for the real scallop and from 4.02 to 16.9 N for the plant-based scallop after grilling, while the chewiness increased from 1.01 to 15.4 for the real scallops and from 3.74 to 13.0 for the scallop analogs. For the real scallops, this effect can be attributed to unfolding and crosslinking of the protein molecules, as well as to moisture loss caused by the high temperature used during grilling, which increased the protein concentration and therefore the gel strength. For the plant-based scallops, the pea proteins were already thermally denatured prior to grilling, but the gel strength may still have increased due to the increase in protein concentration due to heat-induced moisture loss, as well as an increase in protein crosslinking caused by cooking. Interestingly, the relative increases in hardness and chewiness after grilling were greater for the real scallop (13- and 15-fold, respectively) than for the scallop analog (4.2- and 3.5-fold, respectively). This was one of the main reasons that 0.5% pectin was included within the scallop analogs (even though it led to harder gels before cooking). There was an increase in the cohesion of the real scallop after grilling (from 0.72 to 0.82) but a decrease for the scallop analogs (from 0.97 to 0.82), which suggests that cooking had different effects on their abilities to retain their shape after compression. Both the real scallop (96.4%) and scallop analog (93.6%) retained their high degree of springiness after grilling.

**Figure 8:**
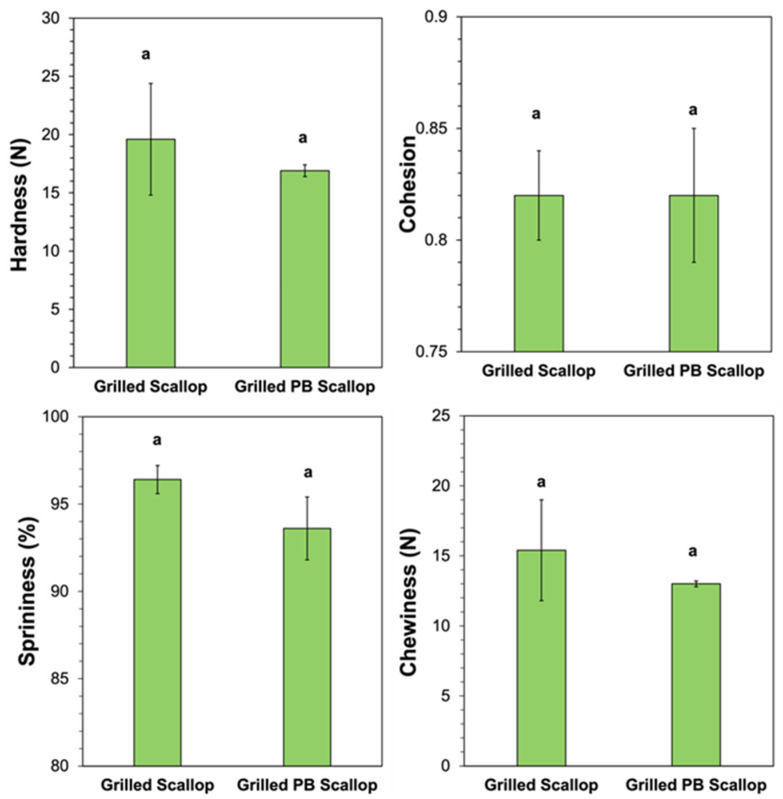
Textual profile analysis of scallop and scallop analog. The scallop analogs contained 10% pea protein, 0.5% pectin, and 2% transglutaminase. Error bars represent the standard errors (*n* = 3), and similar letters mean no statistical difference between treatments (*p* ≤ 0.05).

A direct comparison of the hardness, cohesion, springiness, and chewiness of the grilled real scallop and scallop analog showed they were not statistically different (Figure 8), which suggests their textural properties were similar. Nevertheless, further studies are still required to assess their mouthfeels and textures using sensory studies.

## 4. Conclusions

In summary, we have shown that a soft matter physics approach can be used to produce scallop analogs based on thermal denaturation, phase separation, shearing, and enzymatic gelling of plant protein/polysaccharide mixtures under controlled conditions. Unlike conventional extrusion or shear-cell technologies, no specialized equipment (e.g., an extruder or pressurized high shear cell) is required to create the seafood analogs. The microstructure and physical properties of the scallop analogs could be controlled by adding different pectin concentrations. The gel strength increased upon the addition of a relatively low pectin concentration (0.5%, *w*/*w*) but decreased upon the addition of a higher concentration (1.0%, *w*/*w*). After grilling, the appearance and textural properties of the scallop analogs were very similar to that of the real scallop. Our results suggest that the method developed in this study may prove to be a simple and affordable means of producing plant-based seafood analogs. Nevertheless, further research is still required to fortify the seafood analogs with other nutrients, such as omega-3 fatty acids, vitamins, and minerals, as well as to test their sensory attributes using human studies. Numerous consumer studies have shown that the taste of food products is the main driver for consumer acceptance. Consequently, it will be important to compare the sensory attributes of the plant-based scallops developed in this study with those of real scallops, as well as to establish consumer acceptance and liking of these products. In future studies, we therefore intend to carry out this kind of sensory analysis.

## Figures and Tables

**Table 1:** Colorimetric analysis of scallops and scallop analogs after grilling. The scallop analogs contained 10% pea protein, 0.5% pectin, and 2% transglutaminase. Error bars represent the standard errors (*n* = 3), and similar letters mean no statistical difference between treatments (*p* ≤ 0.05).

	L	a	b
Scallop	52.5 ± 0.9 ^a^	6.2 ± 0.8 ^a^	26.3 ± 0.4 ^a^
Scallop Analog	54.9 ± 2.2 ^a^	6.5 ± 0.9 ^a^	24.0 ± 2.0 ^a^

## Data Availability

The data that support the findings of this study are available from the corresponding author upon reasonable request.
